# Unusual patterns of Monteggia fracture-dislocation

**DOI:** 10.1186/1749-799X-1-12

**Published:** 2006-11-03

**Authors:** Constantinos J Kazakos, Vasilios G Galanis, Dennis-Alexander J Verettas, Alexandra Dimitrakopoulou, Alexandros Polychronidis, Constantinos Simopoulos

**Affiliations:** 1 Department of Orthopaedics, University Hospital of Alexandroupolis, Democritus University of Thrace, Alexandroupolis, Greece; 2 Second Department of Surgery, University Hospital of Alexandroupolis, Democritus University of Thrace, Alexandroupolis, Greece

## Abstract

**Background:**

High-energy trauma may result in uncommon open injuries around the elbow joint. The management of these injuries can be difficult.

**Case description:**

Fourteen patients were treated between 1999 and 2003 and their injuries consisted of Monteggia fracture-dislocations combined with segmental fractures of the ulna or fractures of the forearm bones and/or various more complex trauma such as neural injuries, bone comminution and severe soft tissue injuries around the elbow. Eight of them (57%) were multiply injured with severe additional injuries. All patients underwent surgery within first 4–6 hours. Internal fixation, external fixation or a combination of both methods were used to stabilize fractures while open wounds had secondary closure.

**Results:**

Additional operations were required in 6 patients. The functional results according to the Mayo Elbow Performance Index were excellent or good in eleven patients, and fair or poor in the remaining three. The patients with fair and poor results had suffered from severe neural and soft tissue trauma and/or multiple fractures of the upper extremity.

**Conclusion:**

These injuries should be treated as an emergency. The surgeon should apply any available method that can provide stability to the bone fragments and safe handling of the soft tissues giving priority to internal fixation of the fractures. Severe osseous, soft tissue and neural trauma affect the functional results of the elbow region.

## Background

The term complex joint trauma is used to describe severe injuries that include two or more structural elements of the joint, namely the articulating bones, the major ligaments, the local enveloping soft tissue and the neurovascular structures [1]. Such complex injuries around the elbow joint are often the result of high-energy trauma. They are frequently open. Regel et al [2] defined a complex injury of the elbow joint as a fracture and/or dislocation of the elbow in association with multiple other fractures of the upper extremity, or a severe soft tissue trauma, or a concomitant injury to vessels or nerves. These injuries are uncommon and their management can be difficult [3,4]. Their treatment differs from that of simple fractures because standardized methods cannot be readily employed [5]. This study describes the management of unusual patterns of open complex Monteggia type injuries of the elbow applied over a period of five years in the Orthopaedic Department of Alexandroupolis University General Hospital.

## Case description

Fourteen patients with unusual patterns of Monteggia fracture-dislocation were treated surgically from 1999 to 2003. Eleven were men and 3 women. Their age ranged from 19 to 64 years (average 36). The causes were road traffic accident (8), falls from a height (4) and industrial accidents (2). Eight patients were multiply injured, the injury severity score (ISS) [6,7] ranging from 22 to 41 (average 30) and were admitted in the Intensive Care Unit. The most frequent additional injuries were head injury (8), chest injury (4), abdominal injury (3), femoral fracture (2), acetabular fracture (2) and multiple fractures of the foot (1).

From all fourteen patients there were 7 Monteggia fracture-dislocations with additional (segmental) fracture of the ulnar diaphysis; 4 were type I, 2 were type II and one was type III according to Bado classification [8]. Three were complex patterns of Monteggia fracture-dislocations with additional comminuted fractures of the distal end of both forearm bones; one was type I and 2 were type III according to Bado classification. Two patients had Monteggia fracture-dislocation with additional fractures of the diaphysis of both forearm bones (both were Bado type I). Finally two patients had a Monteggia fracture-dislocation (one Bado type I and one Bado type II) and multiple other fractures of the upper arm.

Open fractures were classified according to Gustilo [9,10]. There were 6 patients with type II, 5 with type IIIA and 3 with type IIIB. There was marked comminution of the fractures (commonly the olecranon and proximal ulnar metaphysis) in 7 patients.

On admission, neural injuries were found in 6 patients. In three the ulnar nerve was involved, in one the posterior interosseous nerve, in one the radial nerve and one patient had the entire brachial plexus injured (Table 1).

**Table 1:** Characteristics of the 14 patients in this study

No	Age/Sex	Skeletal injury	Nerve injury	Management	Outcome/functional results
1	19 M	Mont+segm ulna, II	-	plates	Un/excel
2	30 F	Mont+distal rad-ulna, II	-	plates	Un/excel
3	19 M	Mont+segm ulna, II	brachial plexus	plates	Un/poor
4	23 M	Mont+segm ulna, II	-	plates	N-un/excel
5	36 M	Mont+rad-ulna diaphysis, IIIA	-	plates	Un/excel
6	31 M	Mont+segm ulna, II	posterior interosseous	plates	N-un/good
7	42 M	Mont+segm ulna, II	-	plates	Un/good
8	45 M	Mont+distal rad-ulna, IIIA	ulnar	plates	Un/good
9	21 M	Mont+rad-ulna diaphysis, IIIA	-	plates	Un/good
10	27 F	Mont+distal rad-ulna, IIIB	-	Ex-fix	Un/excel
11	30 F	Mont+segm ulna, IIIA	-	plates	Un/good
12	64 M	Mont+segm ulna, IIIB	ulnar	Ex-fix	Un/fair
13	59 M	Mult-fract, IIIA	ulnar	Ex-fix	N-un/poor
14	57 M	Mult-fract, IIIB	radial	Ex-fix	Un/good

*M*: male, *F*: female, *Mont*: fracture-dislocation Monteggia, *segm*: segmental, *rad*: radius, *Mult-fract*: multiple fractures of the upper extremity, *Ex-fix*: external fixation, *Un*: union, *N-un*: nonunion, *excel*: excellent

Three patients had absent peripheral pulses in the arm on admission.

All patients were operated on within first 4–6 hours of admission. Serious life-threatening injuries were managed first. Initial care of open fractures consisted of irrigation, debridement and wound exploration, reconstruction of ligaments and tendons whenever needed and antibiotic prophylaxis.

Fractures were stabilized by plates in 6 patients with open fractures type II and in 4 patients with open fractures type IIIA. A combination of K-wires and external fixation or external fixation alone was used in 3 patients with open fractures type IIIB and in one patient with open fracture type IIIA. In multiple fractures of the upper arm, all concomitant fractures were operated on primarily, using internal fixation or a combination of internal and external fixation. Two patients underwent radial head resection because of severe comminution.

All wounds primarily were left open. Wound closure was obtained 4–7 days post-injury in 11 patients and in 3 split skin grafts were applied on average 3 weeks post-injury.

The external fixators whenever applied were removed 6–8 weeks after their application and active motion was encouraged. All Kirchner wires were removed at 6–7 weeks.

In addition to the estimation of the range of movements of the elbow, the functional results of the elbow joint were assessed according to the Mayo Elbow Performance Index [11]. This elbow-scoring system evaluates pain (0–45 points), motion (5–20 points), stability (0–10 points) and function (5–25 points). According to this system functional results may be excellent (score>90), good (score 75–89), fair (60–74) or poor (score<60).

## Results

Follow-up ranged from 15 to 58 months (average 34).

Three patients developed non union of the fractures of the ulna and were treated with new osteosynthesis and iliac bone grafts. In the remaining 11 patients the fractures united uneventfully.

All patients with nerve injuries recovered completely within 4 months except one patient with an ulnar nerve injury with segmental loss who had permanent paralysis despite nerve grafts, and another patient with complete brachial plexus lesion who never recovered any function of the arm despite nerve grafts.

The two patients with absent peripheral pulses on admission recovered completely after reduction and stabilization of the fractures. Exploration of the brachial artery revealed no tears or other pathology. The remaining third patient had a tear of brachial artery needed repair with end-to-end anastomosis without postoperative complications.

Three patients developed superficial wound infection which settled with surgical debridement and antibiotics, while 2 more patients developed pin tract infection of their external fixators which settled uneventfully after antibiotic administration.

**Figure 1:**
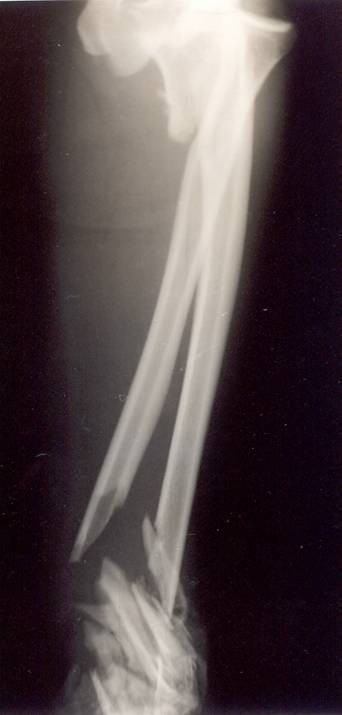
Radiographs of a 45-year-old man multiply injured who had an open complex injury of his left elbow and an ulnar nerve injury after a road traffic accident. Anteroposterior radiographs show a Monteggia fracture dislocation of the left upper arm with additional comminuted fractures of the distal end of both radius and ulna.

**Figure 2:**
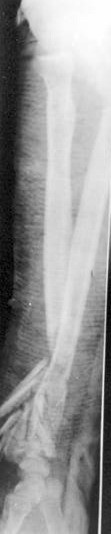
Lateral radiographs of his left forearm and wrist revealed the describing injury.

**Figure 3:**
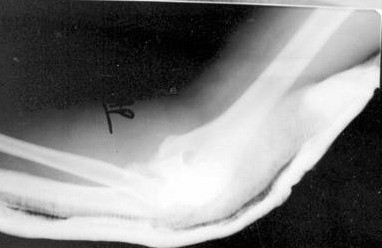
Lateral radiographs of his left elbow showed the Monteggia fracture dislocation.

**Figure 4:**
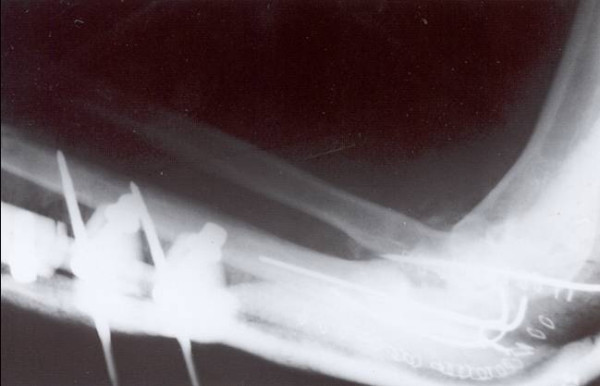
Internal fixation of the open ulnar fracture, reduction of the radial head dislocation.

**Figure 5:**
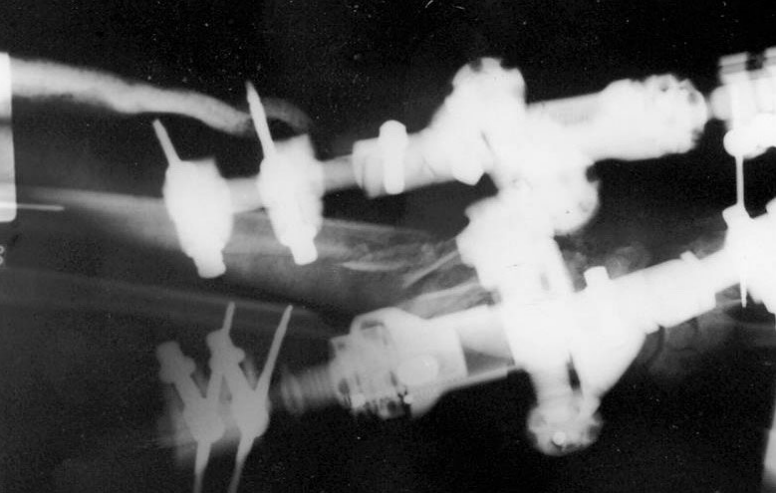
Stabilization of distal forearm fractures by external fixation.

**Figure 6:**
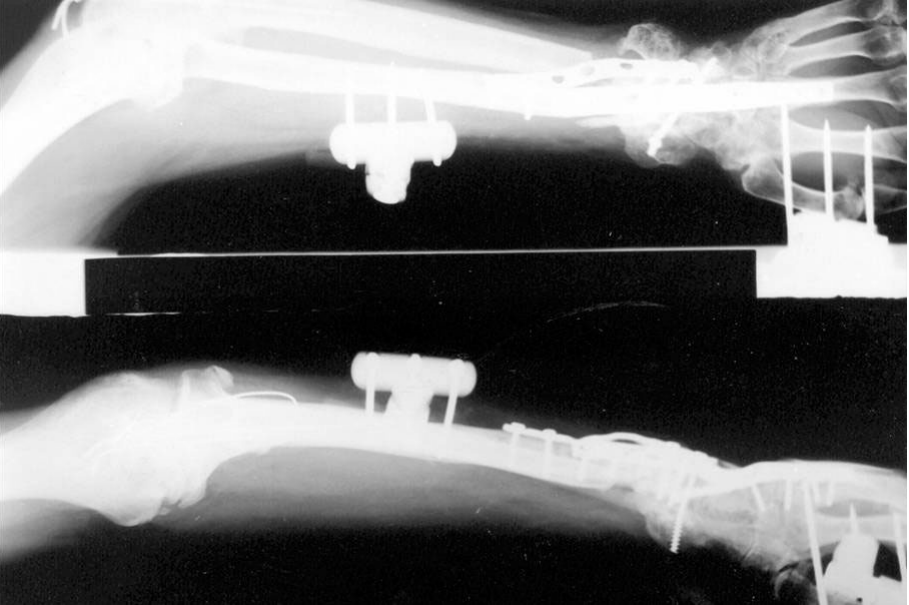
The fractures in the elbow region united and the patient had a good result according to Mayo Elbow Performance Index after 20 months from injury (with a complete recovery of the ulnar nerve the third month from injury). However due to persistent wrist instability and pain he underwent later a carpal arthrodesis.

One patient with a Monteggia fracture-dislocation combined with fractures of distal forearm bones needed after 2 years a carpal arthrodesis due to persistent wrist instability and pain (figure 1, 2, 3, 4, 5, 6).

According to the Mayo Elbow Performance Index, five patients (36%) had excellent result, 6 patients (43%) had good result, 1 patient (7%) had a fair result and 2 patients (14%) had a poor result. Elbow flexion ranged from 60 to 130 deg (average 90). Ten patients developed an extension deficit between 10 – 40 deg. Pronation and supination averaged 70 degrees.

Two patients with excision of the radial head developed moderate instability.

## Discussion

Complex injuries of the elbow can have numerous patterns. They may include fractures at multiple levels in the same bone, or multiple fractures involving several different bones in the upper limb [4,12]. Often these injuries have an open fracture. The patients included in our study had suffered from open Monteggia fracture-dislocations combined with severe trauma around the elbow and/or neural injury and/or other fractures of upper extremity. In our patients, a fracture of the ulna at multiple levels was the most frequent fracture in combination with dislocation of the head of radius and marked comminution of the olecranon or proximal ulna. Open fractures and severe soft tissue injuries were localized more frequently in the ulna.

Stable internal fixation should be the goal of treatment so that early mobilization and physiotherapy can be initiated [4,13,14]. On the other hand, external fixation of open fractures of the elbow has specific limited indications, such as marked fracture comminution, bone loss or extensive soft tissue damage [15-17]. Furthermore, external fixation of the elbow joint can be applied in cases of multiple life threatening injuries and in-patients where the achievement of stable internal fixation is impossible [2,18]. In this series, internal fixation alone was used in all patients with type II open fractures and in 80% of the patients with type IIIA open fractures. The remaining patients had their elbows stabilized with either external fixation alone or with combination of minimal internal fixation (K-wires) and external fixation. The choice for this method in our patients was based on the presence of severe soft tissue damage, instability of the elbow due to ligamentous injury or severe bone comminution, and the general medical condition of the patient. A rigid unilateral external fixator was used in all cases, as opposed to the dynamic fixator preffered by certain authors for early mobilization [3,19,20].

In our series there were 3 patients with non-union of the multiple ulnar fractures. This is in accordance to Wild et al [18] who by using external fixation in the management of massive upper extremity trauma achieved primary bone union in 5 of 16 patients. Ten out of their 16 patients required secondary operation to obtain union because of delayed union or nonunion. Similarily Rogers et al [21] treated 19 patients with concomitant ipsilateral fractures of the humerus and forearm and had 8 cases of non-union.

Early coverage of the open wounds about the elbow by flaps or skin grafts is recommended in order to provide wound closure, decrease infection and tissue oedema and allow early mobilization of the elbow joint [14,22]. On the contrary Tscherne and Regel [23] believe that early relative hypoxia especially of the multiply injured patients has the potential to delay soft tissue healing and provides a susceptibility to infection. In our series 3 patients were treated with split skin grafts within 3 weeks from injury and the rest had delayed closure of their wounds in within 4–7 days. With this method there were only 3 superficial infections that healed after debridement and antibiotic treatment without sequelae.

Neurovascular injuries are common in those serious injuries [15,24]. Pierce and Hodurski [24] in 21 cases of fractures of the humerus, radius and ulna in the same extremity found nerve damage in over 50% of their cases. Regel et al [2] treated 224 complex injuries of the elbow region with 82% of them being open and they had 63,5 % neural injuries, out of which the radial nerve was injured more commonly (42,5%), followed by the brachial plexus (32,5%), the ulnar nerve(22,5%) and the median nerve (2,5%). In our patients ulnar nerve injuries were the most common.

The two patients with absent peripheral pulses had no arterial pathology on exploration and a normal flow was noted after reduction and stabilization of the fractures. A brachial artery tear was found in the third patient that required repair. Although Regel et al [2] noted that compartment syndrome can be a rather frequent vascular complication, in this series, no patient developed this syndrome.

Open complex injuries of the elbow may result in functional deficits of the joint [4,15,25]. Levin et al [15] treated 25 patients with severe grade III upper extremity injuries and had 32 % excellent and good results and 68% fair and poor. Smith and Cooney [17] treated 40 patients with high-energy upper extremity injuries involving the humerus and forearm bones and had 73% good and excellent results, using immediate external fixation, open wound treatment, delayed bone grafting and late internal fixation. In our study 11 patients (79 %) had excellent and good results according to the Mayo Elbow Performance Index. Nine of these patients were treated by internal fixation and 2 by external fixation as their primary treatment. 3 patients (21 %) had a fair or poor result, 2 of them were treated by external fixation and only one by internal fixation as their primary treatment. In addition these patients had serious bone and soft tissue injuries, multiple fractures of the arm and neural lesions. Out of the two patients with poor results one had a permanent ulnar nerve lesion and the other a complete brachial plexus lesion.

Secondary operations are frequently needed in these complex injuries, because it is difficult to obtain a definitive primary treatment. Regel et al [2] noted in patients with multiple injuries (ISS > 30) that primary treatment was not possible in 37% of patients. In our study in 6 patients (43%) secondary operations were required.

## Conclusion

Open complex injuries of the elbow may defy the classical principles of fracture treatment and the surgeon should apply any available method that can provide stability to the bone fragments and safe handling of the soft tissues giving priority to internal fixation of the fractures.

Despite the fact that at least half of these patients are multiply injured, treatment should be initiated as soon as possible. Severe bone loss, serial osseous injuries and neural lesions may affect the final functional results of the elbow joint.

## Competing interests

The author(s) declare that they have no competing interests.

## Authors' contributions

CK conceived of the study, and participated in its design and coordination and helped to draft the manuscript.

VG conceived of the study, and participated in its design and helped to draft the manuscript.

DV conceived of the study, and participated in its design and coordination and helped to draft the manuscript.

AD helped to draft the manuscript.

AP helped to draft the manuscript.

CS participated in its design and coordination and helped to draft the manuscript.

All authors read and approved the final manuscript.
